# People with early-onset colorectal cancer describe primary care barriers to timely diagnosis: a mixed-methods study of web-based patient reports in the United Kingdom, Australia and New Zealand

**DOI:** 10.1186/s12875-023-01967-0

**Published:** 2023-01-14

**Authors:** Klay Lamprell, Diana Fajardo Pulido, Gaston Arnolda, Bróna Nic Giolla Easpaig, Yvonne Tran, Syeda Somyyah Owais, Winston Liauw, Jeffrey Braithwaite

**Affiliations:** 1 Australian Institute of Health Innovation, Macquarie University, North Ryde, NSW 2109 Australia; 2 Pontificia Universidad Javeriana, Bogotá, DC Colombia; 3 College of Nursing and Midwifery, Charles Darwin University, Darwin, NT Australia; 4 Macquarie University Hearing, Macquarie University, North Ryde, NSW Australia; 5 St. George Cancer Care Centre, St. George Hospital, Kogarah, NSW Australia; 6 St. George Hospital Clinical School, University of New South Wales, Sydney, NSW Australia

**Keywords:** Early-onset colorectal cancer, Primary care, GP, Patient experience, Mixed methods, Patient-reported, Qualitative

## Abstract

**Background:**

People with early-onset colorectal cancer, under the age of 50, are more likely to experience diagnostic delay and to be diagnosed at later stages of the disease than older people. Advanced stage diagnosis potentially requires invasive therapeutic management at a time of life when these patients are establishing intimate relationships, raising families, building careers and laying foundations for financial stability. Barriers to timely diagnosis at primary care level have been identified but the patient perspective has not been investigated.

**Methods:**

Personal accounts of cancer care are increasingly accessed as rich sources of patient experience data. This study uses mixed methods, incorporating quantitative content analysis and qualitative thematic analysis, to investigate patients’ accounts of early-onset colorectal cancer diagnosis published on prominent bowel cancer support websites in the United Kingdom, Australia and New Zealand.

**Results:**

Patients’ perceptions (*n* = 273) of diagnostic barriers at primary care level were thematically similar across the three countries. Patients perceived that GPs’ low suspicion of cancer due to age under 50 contributed to delays. Patients reported that their GPs seemed unaware of early-onset colorectal cancer and that they were not offered screening for colorectal cancer even when ‘red flag’ symptoms were present. Patients described experiences of inadequate information continuity within GP practices and across primary, specialist and tertiary levels of care, which they perceived contributed to diagnostic delay. Patients also reported tensions with GPs over the patient-centredness of care, describing discord related to symptom seriousness and lack of shared decision-making.

**Conclusions:**

Wider dissemination of information about early-onset colorectal cancer at primary care level is imperative given the increasing incidence of the disease, the frequency of diagnostic delay, the rates of late-stage diagnosis and the dissatisfaction with patient experience reported by patients whose diagnosis is delayed. Patient education about diagnostic protocols may help to pre-empt or resolve tensions between GPs’ enactment of value-based care and patients’ concerns about cancer. The challenges of diagnosing early-onset colorectal cancer are significant and will become more pressing for GPs, who will usually be the first point of access to a health system for this growing patient population.

**Supplementary Information:**

The online version contains supplementary material available at 10.1186/s12875-023-01967-0.

## Introduction

“If I hadn’t ignored my symptoms, if I had gone to my GP when things changed and had symptoms that were unusual for me, maybe it would’ve been caught earlier …. Maybe it wouldn’t have though, because who gets bowel cancer at age 33?”

The global incidence of colorectal cancer (CRC) in people aged under 50 is rising rapidly, in contrast to stable or declining rates of CRC in older adults [1–6]. CRC occurring before the age of 50, known as early-onset colorectal cancer (EoCRC), currently accounts for just over 6% of all CRC cases annually in the United Kingdom (UK), [7] some 10% of annual CRC incidence in Australia [8] and just over 11% of all CRC cases annually in New Zealand (NZ) [9]. The average increase in early-onset diagnoses (aged 20–49 years) over a ten year period was 4% per annum in NZ (2007–2016) and 2.8% per annum in both Australia (2006–2015) and the UK (2003–2012); over the same periods, the incidence in CRC diagnoses in those aged 50 and above was decreasing in NZ and Australia, and unchanged in the UK [4]. As a result of increasing EoCRC and static or decreasing CRC in older patients, people under the age of 50 are projected to become a larger proportion of all CRC patients, especially in high-income countries [1, 4].

EoCRC takes longer to diagnose than traditional CRC [10, 11] and features a greater likelihood of diagnosis in late stages of the disease [1, 3, 10, 12]. Late stage diagnosis in EoCRC does not appear to impact mortality but escalates the prospect of invasive therapeutic management [13] at a time of life when these patients are establishing intimate relationships, raising families, building careers and laying foundations for financial stability [14]. Timely diagnosis is crucial to this growing patient population [10, 15].

Data on healthcare-led barriers to timely EoCRC diagnosis [15–17] indicate that people eventually diagnosed with EoCRC are likely to spend three months to five years seeking a diagnosis and may have between three and eleven visits to their general practitioners (GPs) [10, 18, 19] and may also see up to four GPs in pursuit of a diagnosis for their symptoms [19]. Patients report also, to a lesser extent, multiple visits to specialists and to emergency services as part of the trajectory for EoCRC diagnosis [17].

Current knowledge of diagnostic delay in EoCRC relies on surveys of EoCRC patient populations and quantitative analysis of patient records. As yet we have little understanding of how EoCRC patients perceive the quality of care they receive during their diagnostic phase and what they think about the actions of their GPs. This information will be of value in understanding the needs of this newly emerging patient population.

We present our investigation of online patient reports of EoCRC diagnosis. We asked: *What experiences and perceptions of health service barriers to diagnosis are reported by EoCRC patients in their online accounts of diagnosis? How do EoCRC patients who have experienced diagnostic delay characterise their interactions with GP services?*

## Methods

### Approach

Personal accounts of healthcare experiences published online are increasingly accessed as rich sources of patient-reported data [20, 21]. There are methodological challenges related to collecting data from these patients' accounts, including ethical concerns, sampling issues and matters of validity [22]. In this section we describe our mixed methods approach to addressing these challenges in order to ensure rigour and transparency [23, 24].

### Researcher reflexivity

The study was designed and led by an experienced qualitative researcher with skills in the analysis of web-based patient accounts of personal experience. The research team comprised highly experienced researchers across clinical practice, social science, health systems research and qualitative and quantitative methods. Team members shared interests in identifying, characterising and responding to real-world complexities in the delivery of timely diagnosis to the newly emerging population of people with EoCRC.

### Population and context

Websites hosted by three key charitable support organisations were selected as specific research settings: Bowel Cancer Australia, [8] Bowel Cancer UK [7] and Bowel Cancer New Zealand [8]. Australia, New Zealand (NZ) and the United Kingdom (UK) were selected because CRC care is comparable in being financially integrated at primary care level, whereas other anglophone countries, such as the United States, feature highly varied levels of integrated primary healthcare. With permission from these organisations we accessed the public domain sections of these websites in which bowel cancer survivors ‘post’ accounts of their experiences, under banners such as ‘real life stories’ or ‘your stories’ [25].

The study population was selected by criteria sampling rather than representative sampling. Personal accounts were excluded from the study if they were not written by people who were diagnosed under 50 years of age or if accounts solely comprised feedback on, or criticism of, a named institution or clinician.

### Ethics and patient and public involvement (PPI)

The study was granted ethical and scientific approval by the Macquarie University Human Research Ethics Committee (MQ HREC Reference No:52020666115757). The committee was satisfied that the use of the personal accounts as data matched the expectations of the individuals who published their accounts online in public domain sections of prominent cancer support websites.

### Data collection

Eligible personal accounts of EoCRC published on the host websites as at February 2021 were collected as data. Each account was processed as a separate research text and downloaded into the NVivo 12 qualitative data analysis software program (QSR International Pty Ltd. Version 12, 2018). Each text was de-identified and attributed a unique participant study number to be used on all study documents.

### Data analysis

Our mixed methods analysis of the personal accounts involved: quantitative description of the demographic and health status characteristics of the study population; quantitative content analysis to understand the frequency of topics and events of interest to the study; and qualitative thematic analysis to examine the descriptions and perceptions of the events that people reported in their narrative accounts.

The qualitative analysis focused on three key categories of patient experience that are assessed in patient-reported experience measures (PREMs): clinical assessment; continuity of care; and interpersonal care. Sentence-by-sentence analysis organised parts of each text into these three categories. We then looked for topics and themes of EoCRC patient experience of diagnosis within each of these categories. One researcher led the thematic analysis with iterative review and consensus from co-authors.

### Techniques to enhance trustworthiness

Though final decisions on themes were not based on numbers of references, cross-checking against the quantitative results was a useful mechanism for comprehending undue emphasis on isolated experiences or experiences only of interest to individual researchers. In particular, the quantitative results provided an important validating context for understanding the scope of negative and dissatisfying experiences comprised in the texts, given that the personal accounts also comprised reports of positive experiences and timely diagnosis.

## Results

### Quantitative analysis

#### Demographic and clinical characteristics

Across the three websites, 273 personal accounts met the inclusion criteria: 136 (50%) from UK, 116 (42%) from Australia and 21 (8%) from NZ. Over two-thirds were female (73%), and almost half were aged 30 to 39 years (48%). Two-thirds reported being married or in a relationship (66%) and over half reported being pregnant or having children under 18 years (55%), with the remainder not reporting.

Overwhelmingly, the narrators reported a diagnosis of bowel or colon cancer (94%), with only 6% indicating rectal cancer. The majority was diagnosed at an advanced stage (III or IV) (66.3% overall). When describing the status of their health, 22% gave no indication, 20% were in treatment, 42% were in complete remission, and 4% reported progression. For the most part comorbidities, family history and genetic mutations were not mentioned in the narratives, but these were recorded by 7, 16 and 9% of narrators, respectively. As shown in Table 1, countries sometimes varied from the overall pattern described above.

**Table 1:** Demographic, clinical and treatment characteristics described in early-onset colorectal cancer patients’ personal narratives published online in Australia, NZ and United Kingdom

Categories		Australia (***n*** = 116)		NZ (***n*** = 21)		UK (***n*** = 136)		Total (***n*** = 273)	
		***n***	%	***n***	%	***n***	%	***n***	%
**Demographic**	**Gender**								
	Female	92	79.3	14	66.7	94	69.1	200	73.3
	Male	21	18.1	7	33.3	38	27.9	66	24.2
	Not reported	3	2.6	0	0.0	4	2.9	7	2.6
	**Relationship status**								
	Married or in relationship	77	66.4	6	28.6	98	72.1	181	66.3
	Single	35	30.2	15	71.4	37	27.2	87	31.9
	Not reported	4	3.5	0	0.0	1	0.7	5	1.8
	**Children**								
	Pregnant or under 18 yrs	60	51.7	6	28.6	83	61.0	149	54.6
	Not reported	56	48.3	15	71.4	53	39.0	124	45.4
	**Age at diagnosis**								
	20–29 yrs	19	16.3	3	14.3	12	8.8	34	12.5
	30–39 yrs	57	49.1	15	71.4	59	43.4	131	48.0
	40–49 yrs	29	24.0	2	9.5	28	20.6	59	21.6
	Not specifically reported	11	10.6	1	4.8	37	27.2	49	18.0
**Clinical**	**Cancer type**								
	Bowel or colon	108	93.1	19	90.5	130	95.6	257	94.1
	Rectal	8	6.9	2	9.5	6	4.4	16	5.9
	**Diagnosis stage**								
	Less advanced (Stage I-II)	14	12.1	1	4.8	26	19.1	41	15.0
	Advanced (Stage III-IV)	70	60.3	17	81.0	94	69.1	181	66.3
	Not reported	32	27.6	3	14.3	16	11.8	51	18.7
	**Current diagnosis**								
	Disease in progression	4	3.5	0	0.0	7	5.2	11	4.0
	Complete remission	45	38.8	5	23.8	64	47.1	114	41.8
	Partial remission	15	12.9	0	0.0	8	5.9	23	8.4
	In treatment	21	18.1	2	9.5	32	23.5	55	20.2
	Stable	5	4.3	1	4.8	3	2.2	9	3.3
	Not reported	26	22.4	13	61.9	22	16.2	61	22.3
	**Family history**								
	Yes	23	19.8	2	9.5	20	14.7	45	16.5
	No	8	6.9	4	19.1	7	5.2	19	7.0
	Not reported	85	73.3	15	71.4	109	80.2	209	76.6
	**Genetic mutation**								
	Yes	6	5.2	3	14.3	16	11.8	25	9.2
	No	2	1.7	0	0.0	2	1.5	4	1.5
	Not reported	108	93.1	18	85.7	118	86.8	244	89.4

#### Diagnostic journey

Table 2 summarises aspects of the diagnostic journey reported by patients in each country. The most common symptom was abdominal pain (49%) followed by stool change (31.5%), rectal bleeding (30%), fatigue (29%) and bowel habit change (29%). The first consult was usually a routine primary care visit (73% of narratives) or an emergency primary care or hospital visit (13%). There was a variety of initial diagnoses; the most common were irritable bowel syndrome (17%), haemorrhoids (9%), anaemia (8%), inflammatory bowel disease (7%) and gynaecological problems (7%). Narratives mentioned a number of diagnostic tests, most commonly colonoscopy (70%), CT scan (45%) and MRI (17%); in 11% of narratives the patient initiated the test request.

**Table 2:** Journey from first help-seeking to diagnosis described in early-onset colorectal cancer patients’ personal narratives published online in Australia, New Zealand and United Kingdom

Categories	Australia (***n*** = 116)		NZ (***n*** = 21)		UK (***n*** = 136)		Total (***n*** = 273)	
	***n***	%	***n***	%	***n***	%	***N***	%
**Symptoms (multiple possible)**								
Abdominal pain	56	48.3	13	61.9	66	48.5	135	49.5
Stool change	34	29.3	7	33.3	45	33.1	86	31.5
Fatigue	30	25.9	8	38.1	42	30.9	80	29.3
Rectal bleeding	29	25.0	3	14.3	49	36.0	81	29.7
Bowel habit change	27	23.3	9	42.9	44	32.4	80	29.3
Bloating	21	18.1	3	14.3	22	16.2	46	16.9
Nausea or vomiting	13	11.2	6	28.6	15	11.0	34	12.5
Weight change	10	8.6	3	14.3	21	15.4	34	12.5
Other	27	23.3	5	23.8	8	5.9	40	14.7
None	5	4.3	0	0.0	3	2.2	8	2.9
**Initial diagnosis (Multiple possible)**								
Anaemia or iron deficiency	14	12.1	0	0.0	7	5.2	21	7.7
Appendicitis, coeliac dis. or diverticulitis	12	10.3	1	4.8	4	2.9	17	6.2
Irritable Bowel Syndrome	10	8.6	2	9.5	35	25.7	47	17.2
Haemorrhoids	9	7.8	1	4.8	15	11.0	25	9.2
Inflammatory Bowel Disease	8	6.9	1	4.8	11	8.1	20	7.3
Gynaecological	6	5.2	3	14.3	10	7.4	19	7.0
Diet related changes	5	4.3	2	9.5	5	3.7	12	4.4
Viral infection	5	4.3	4	19.1	5	3.7	14	5.1
Other	18	15.5	2	9.5	8	5.9	28	10.3
**Diagnostic test (Multiple possible)**								
Colonoscopy	82	70.7	14	66.7	96	70.6	192	70.3
CT scan	56	48.3	4	19.1	63	46.3	123	45.1
MRI	18	15.5	1	4.8	27	19.9	46	16.9
PET	13	11.2	0	0.0	6	4.4	19	7.0
Ultrasound	11	9.5	2	9.5	11	8.1	24	8.8
Other	10	8.6	2	9.5	9	6.6	21	7.7
X-Ray	5	4.3	0	0.0	1	0.7	6	2.2
Sigmoidoscopy	4	3.5	0	0.0	16	11.8	20	7.3
Faecal	1	0.9	2	9.5	1	0.7	4	1.5
**Time from initial symptoms to first consult**								
Less than three months	43	37.1	7	33.3	43	31.6	93	34.1
3 to 12 months	29	25.0	2	9.5	25	18.4	56	20.5
More than 12 months	8	6.9	2	9.5	8	5.9	18	6.6
Not specifically reported	36	31.0	10	47.6	60	44.1	106	38.8
**Type of first consult**								
Primary care - Routine	76	65.5	15	71.4	109	80.2	200	73.3
Emergency Department	10	8.6	0	0.0	9	6.6	19	7.0
Primary care – Emergency	8	6.9	3	14.3	5	3.7	16	5.9
Not reported	22	19.0	3	14.3	13	9.6	38	13.9
Self-efficacy								
Requested colonoscopy	10	8.6	0	0.0	3	2.2	13	4.8
Requested specific CRC investigation	4	3.5	2	9.5	5	3.7	11	4.0
Requested ultrasound	1	0.9	1	4.8	0	0.0	2	0.7
Requested other tests	3	2.6	0	0.0	1	0.7	4	1.5
Not reported	98	84.5	18	85.7	127	93.4	243	89.0
**Time from first consult to diagnosis**								
Less than three months	36	31.0	2	9.5	19	14.0	57	20.9
3 to 12 months	15	12.9	2	9.5	51	37.5	68	24.9
More than 12 months	11	9.5	4	19.1	26	19.1	41	15.0
Not reported	54	46.6	13	61.9	40	29.4	107	39.2
**Referrals**								
Only 1	44	37.9	2	9.5	25	18.4	71	26.0
Between 2 and 4	14	12.1	2	9.5	9	6.6	25	9.2
More than 5	9	7.8	4	19.1	4	2.9	17	6.2
Not reported	49	42.2	13	61.9	98	72.1	160	58.6
**Number of outpatient visits to diagnosis**								
Only 1	5	4.3	1	4.8	3	2.2	9	3.3
Between 2 and 4	21	18.1	1	4.8	25	18.4	47	17.2
More than 5	5	4.3	4	19.1	30	22.6	39	14.3
Not reported	85	73.3	15	71.4	78	57.4	178	65.2
**Number of inpatient visits to diagnosis**								
Only 1	8	6.9	3	14.3	10	7.4	21	7.7
Between 2 and 4	3	2.6	0	0.0	13	9.6	16	5.9
More than 5	0	0.0	1	4.8	2	1.5	3	1.1
Not reported	105	90.5	17	81.0	111	81.6	233	85.4
**Reason for delay in diagnosis**								
Interval to consult with specialist/test	2	1.7	1	4.8	9	6.6	12	4.4
Missed diagnosis opportunity	20	17.2	8	38.1	38	27.9	66	24.2
Not reported	94	81.0	12	57.1	89	65.4	195	71.4

While the number of specialist referrals from primary care was not mentioned in 59% of narratives, there was only a single referral in 26% of narratives, while around 15% required two or more specialist referrals. Two-thirds of narratives did not mention the number of outpatient visits (66%), but 14% reported 5 or more such visits. Inpatient visits were mentioned in only 15% of narratives, with diagnosis occurring in the first hospitalisations in more than half of these (8%).

The amount of time from first consultation to diagnosis was not mentioned in 39% of narratives; diagnosis took < 3 months in 21%, 3–12 months in 25%, and 1–5 years in 15%. Among the 40% of accounts that mentioned a duration of three or more months from initial consultation to diagnosis, 24% attributed the delay to missed diagnostic opportunity, while 71% did not report any reason.

Once again, there is some variation in issues mentioned by patients in different countries. Possible differences include, for example, that: Australian narratives mentioned anaemia more frequently; NZ narratives less frequently mentioned CT scans or MRIs; Australian and NZ narratives more frequently mentioned that the patient had initiated a request for a diagnostic test (14–15%) compared to UK narratives (7%); and Australian narratives mentioned less time between first consult to diagnoses than NZ narratives, and more frequently mentioned a single specialist referral.

### Qualitative analysis

Unsurprisingly, EoCRC patients’ accounts portrayed help-seeking experiences as satisfactory or positive when diagnosis was straightforward and timely, and characterised care as disappointing or frustrating when barriers to diagnosis were experienced. Our qualitative results suggest that patients experienced similar barriers to timely diagnosis at primary care level in all three countries of interest, despite variations in health systems with regards to clinical practice guidelines (CPGs), referral conventions and diagnostic testing protocols.

In Table 3 we present the key themes that emerged within each of the three categories of patient experience, accompanied by illustrative quotes derived from the qualitative analysis. Additional details on the results in each theme are available in the Additional file 1 Appendix. Low suspicion of cancer given age under 50 was a persistent theme across all stories of healthcare barriers to timely diagnosis, inclusive of GPs, specialists and emergency care services. Patients perceived their age as a factor that shaped the nature of clinical assessments and initial diagnoses, influenced the investigations conducted and referrals given, and created tensions between patients and doctors which obstructed shared decision-making. Eventual referrals for non-urgent colonoscopies and lengthy wait times for non-urgent colonoscopies was also a common theme in descriptions of delayed diagnosis and was reported as a cause of dissatisfaction with GPs.

**Table 3:** Patient-reported barriers to timely diagnosis described in early-onset colorectal cancer patients’ personal narratives published online in Australia, New Zealand and United Kingdom: key themes and illustrative quotes

CATEGORY	THEME	ILLUSTRATIVE QUOTE
1. Clinical assessment	1.1 Health provider response to age < 50	“The biggest challenge I faced as someone diagnosed with bowel cancer under 50 was getting my symptoms taken seriously by doctors, even though my father had had bowel cancer with liver metastases … The occasional blood kept happening, so I spoke to a doctor again. It was again brushed aside as probably being haemorrhoids, but the GP investigated and was unable to see any. I was prescribed suppositories in case there was a haemorrhoid higher up in the rectum... but the intermittent blood in the toilet/in my poo continued, so I told a doctor again. I was informed it was unlikely to be anything sinister because I was my early forties, plus I wasn’t experiencing any change of appetite or pain, or constipation.” F. Age: 43; Stage: not reported
	1.2 Prioritisation of common conditions	“Numerous symptoms led me to that colonoscopy; urgency, frequency, bleeding, bloating, pain etc. I had had these symptoms for years, however they were always explained as something else, ovarian tumours, IBS, back to back pregnancies, difficult deliveries and/or post-natal complications. No one ever suspected Bowel Cancer …” F. Age: 34; Stage III
	1.3 Adequacy of investigations	“I initially had a faecal test which didn’t detect anything, and then a CT Scan showed some swelling in my sigmoid colon and was diagnosed as having Colitis (swelling of the bowel). It apparently presents similar to a tumour on a CT Scan, but a tumour was ruled out given my age.” M. Age:35; Stage: III
2. Continuity of care	2.1 Referral delays	“Eventually, after a good few months, I worked up the courage to go to the GP. She asked me a few questions and told me that I was too young to worry about it being cancer and as I have had a child, it was probably haemorrhoids …. A few months later, with more bleeding I noticed that there was mucus now in my stool and I decided that I wanted to get it looked into. That doctor wouldn’t give me a colonoscopy so I went to a different doctor and was forceful in asking for a referral to a colorectal surgeon to have a colonoscopy.” F. Age 36; Stage III
	2.2 Continuity across multiple providers	“I saw several different doctors in the 16 months at my large and busy GP practice. My persistent symptoms were not connected together and I was essentially ‘lost’ in the system. I’m left with questions about ‘what if’ I was diagnosed earlier.” F. Age: 26; Stage: III
	2.3 Wait times for colonoscopy	“I wasn’t in any pain. In fact the only symptoms I had was sporadic blood showing up in my poop and some constipation and cramps. I’d been doing a bit of international travel with work and so the first doctor I saw thought I might have picked up a bug. I had a stool test but it came up inconclusive. About two months later a second GP thought I might have a cut in the lining of my rectum and sent me home with some cream. Finally, a month later my local GP recommended me for a colonoscopy … I would have had to wait a few months in the public system so I decided to get mine done privately (… ouch). But that ended up being the best decision I ever made.” M: Age: 47; Stage III
3. Interpersonal care	3.1 Tensions over symptom seriousness	“Advertising tells us that if we notice blood when we go to the toilet, we must tell our doctor … I was embarrassed about it but knew that it was necessary to tell the doctor, especially since my father had had bowel cancer. To my surprise, my symptoms were brushed aside as probably being nothing - maybe a scratch or irritation.” F. Age: 43; Stage: not reported
	3.2 Self-efficacy	“I would often get severe stomach cramps and only a foetal position with pressure on the area would help make it go away. I went to the doctors numerous times over the years. But I always left feeling stupid and that it was all in my mind as they could never feel or find anything wrong with me.” F. Age: 31; Stage IV
	3.3 Reassurance referrals	“After numerous visits to him, he referred me to see a gastroenterologist. The gastroenterologist thought that this was due to the reason that I was under a lot of stress, the fact that food intolerances get worse when you are stressed, and the bleeding would eventually stop. Then I got referred to see a dietician. I got given a whole lot of food that I should avoid. I’ve noticed that previously my bleeding would be on and off, but this time, it was there consistently, every day, with a severe pain on the right side of the stomach. I had to see my GP again, I cried at his office, saying I was worried I could have bowel cancer. He reassured that this was nothing serious. Another week went by and I had to see the GP again, tears were pouring down my face as I told him that I thought something was not right with me. I got asked to see the gastroenterologist again, she in fact laughed at me and said that people with my ethnic background, my age and being a female had a very low chance of it. She said she would put me on an elective list for a colonoscopy and that there was no reason to worry.” F. Age: 38; Stage III

## Discussion

### Clinical assessment and barriers to diagnosis

Patients in our study reported that anal bleeding and blood in stools were commonly attributed to haemorrhoids, persistent anaemia was managed as a dietary-related iron deficiency and ongoing symptoms of abdominal pain, bloating, bowel changes and fatigue were investigated and managed for IBS or other common gastrointestinal disorders as well as gynaecological conditions.

A quarter of patients who reported delays of over three months conveyed frustration and dissatisfaction that their GPs had spent so much time focusing on common conditions and described their GP’s low suspicion of cancer as poor-quality care. These findings were largely consistent across the three countries of interest, supporting literature in all three countries that EoCRC patients who experience delays are dissatisfied with the diagnostic actions of their GPs when cancer investigations are not prioritised [2, 10, 15, 17, 26].

This dissatisfaction amongst EoCRC patients points to the need for greater patient education of diagnostic protocols [27]. Though the incidence of CRC is increasing in people aged under 50 years, [4, 13, 28] it is infrequent compared to CRC in older populations. GP inexperience with EoCRC may contribute to diagnostic delay [29] but low suspicion of cancer given age under 50 is also justified. Evidence is emerging of the clinical and molecular features that distinguish EoCRC from traditional CRC, [2, 30, 31] which will drive the evolution of EoCRC-specific CPGs. Until that guidance is made available to GPs, a focus on investigation for common conditions is clinically appropriate in individuals with low or average risk of CRC [31].

A confounding aspect of our data was a seeming gender disbalance in clinical assessment; only female patients eventually diagnosed with EoCRC reported that their GPs diagnosed IBS. This finding may be influenced by the gender disproportion in our study population; while approximately the same number of women as men contract CRC under the age of 50, [28] three quarters of our narrators were female. Nevertheless, the literature indicates that the ratio of women to men with IBS across all patient populations is estimated as 3:1, [32, 33] suggesting that IBS would be diagnosed in at least some of the men in the study sample.

Similarly, only female patients reported that GPs discussed a psycho-emotional diagnosis such as stress, anxiety, and disordered eating. Women may be more likely than men to ask questions and discuss concerns in detail, [34] which may lead GPs to explore the psychosocial implications of ongoing symptoms with women more than with men. Our study, however, is not the first to identify possible gender disparity as a factor in delayed diagnosis of CRC. Siminoff et al. [34] found that women may be more likely to experience a missed opportunity for CRC diagnosis than men [34] and Rogers et al. [35] found that men were more likely than women to experience appropriate clinical outcomes in CRC diagnosis.

### Continuity of care and barriers to diagnosis

Seeing one GP over time has been associated with a marginally higher likelihood of early cancer diagnosis, [36] particularly with cancers featuring generalised symptoms [37]. Our findings support evidence to the contrary; repeated visits to the same GP increased delays in referral of patients for CRC investigations [38–40]. The referral reluctance perceived by patients in our study may indicate that GPs are protecting patients from unnecessary invasive testing and avoiding low-value use of medical resources. Additionally, the referral delay events identifiable in our data may be exaggerated by the demographics of our study population; half the personal accounts were written by people aged 30–39 for whom there is generally less risk of CRC [41, 42].

Aspects of our data, however, challenge this explanation for GPs’ referral decisions. Patients with relevant family history of CRC and persistent symptoms identified as ‘red flags’ [43] for CRC referral at any age [44] reported being given non-urgent referrals when presenting repeatedly to the one GP. These patients described these actions as a result of age bias that delayed diagnosis.

Patients also reported inadequate continuity of care across multiple providers as a barrier to timely diagnosis. Patients described locum GPs, rotating medical staff in large primary care practices, specialist appointments, emergency care for pain management and changing GPs as contexts involving multiple providers.

Poor information-continuity across medical providers is a well-established diagnostic liability, even when changing doctors within one practice [45, 46]. Many patients reported being surprised and frustrated by how little information about their diagnostic trajectory to date was contained in their medical records and in GP referral letters. This points to the broader issue of patients’ expectations that records of their assessments and treatments are adequately shared between health care professionals [46]. Patients in our study voiced concerns that in retelling their histories they would miss vital information and, importantly, would not be able to convey the futility of prior efforts towards diagnosis.

### Interpersonal care and barriers to diagnosis

Issues with interpersonal communication and shared decision-making were prominent in the personal accounts of delayed diagnosis across the three countries. In particular, patients’ concerns about symptom seriousness were sources of tension in interactions and relationships with GPs, but also with providers at all levels of care. The refrain, “I wasn’t taken seriously,” which resounds across these stories, is evident also in surveys of EoCRC patient populations [10, 15].

Symptom seriousness has been identified as a crucial point of patient-doctor divergence in medical consultations [47]. Misalignment between a patient’s concerns about cancer and clinical suspicion of cancer at primary care level is widespread [48]. Patients reported that GPs who were responsive to this tension offered a non-urgent referral for colonoscopy, or for other cancer screening, as an explicit act of discord resolution and reassurance. ‘Reassurance referrals’ have been identified as an appropriate means of satisfying the criteria for patient-centred care [44]. In other responses to this tension over symptom seriousness, patients described turning to avenues of care such as emergency services for pain management, or changing GPs.

Our data are notable for the absence of documented communication between patients and GPs about diagnostic protocols for CRC detection in people under the age of 50. GPs may pre-empt tensions over symptom seriousness through discussion about the rationale for referral decisions [49]*;* this may be especially relevant for patients with low risk of CRC and non-specific symptoms.

## Conclusions

Primary care barriers to timely diagnosis are reported by EoCRC patients in three key areas of patient experience: clinical assessment; continuity of care; and patient-centredness of interpersonal interactions. Perceptions of barriers to timely diagnosis across all three areas may stem in part from patients’ limited understanding of the guidelines that steer diagnostic decision-making. Patients perceive their GPs’ low suspicion of cancer given age under 50 as an age bias that contributes to delays. There may be value for GPs in addressing this perception in discussion with patients and providing patients with knowledge about diagnostic protocols.

We conclude that with the rising global incidence of CRC in people aged under 50, there is a mounting imperative for GPs to receive information and clinical guidance on EoCRC diagnosis. In the absence of EoCRC-specific diagnostic protocols, some sources of diagnostic delay may be unavoidable, especially for low-risk individuals presenting with broad symptoms. Other sources of GP-led delay described by patients, such as failure to respond to red flags and to take note of repetitive help-seeking, are clearly addressable. The challenges of diagnosing CRC in young people are significant and will become more pressing for GPs, who will usually be the first point of access to a health system for this growing patient population.

### Limitations

In providing the demographics represented in the personal accounts, and by presenting the percentages of personal accounts that identify specific barriers in the nominated categories, we have strived for transparency about the limitations of our data. We note, for example, that our data over-represent experiences of younger EoCRC patients and women, and under-represent patients with rectal cancers.

Participants self-selected to write their narratives, a known source of bias, and thus our study is not generalisable. Notably, there is an absence of data specific to culturally and linguistically diverse (CALD) EoCRC populations, given the significant Indigenous populations in two of the settings of interest to the study: Australia and NZ. As a consequence, the results do not reflect evidence in the literature that racial and ethnic factors impact timely CRC diagnosis [50–52]. Language barriers as well as socio-cultural norms and conventions related to personal privacy and autobiographical narration, amongst other factors, may preclude members of migrant and Indigenous populations from publishing online accounts of patient experience or from taking part in interviews and surveys [53]. Developing opportunities for CALD patients to self-report their experiences of EoCRC diagnosis, for example through social media, [53, 54] may enhance the capacity of patient experience research to inform and inspire equitable primary care diagnostic policy and practice [55].

## Supplementary Information

**Additional file 1:** **Appendix.** Detailed results of qualitative analysis.

## Data Availability

The datasets analysed during the current study are publicly available on the websites of Bowel Cancer UK, Bowel Cancer Australia and Bowel Cancer NZ. The datasets analysed during the current study are also available from the corresponding author on reasonable request. Information/data related to the study is stored on password-protected computers and archived on electronic databases on a secure server. The records of this research study will be retained for a minimum of 5 years post study completion or last publication, in accordance with the Australian National Health and Medical Research Council guidelines for management of data and information in research.
