# Supplementary material for: People with early-onset colorectal cancer describe primary care barriers to timely diagnosis: a mixed-methods study of web-based patient reports in the United Kingdom, Australia and New Zealand
Source: BMC Prim Care. 2023 Jan 14;24:12. doi: 10.1186/s12875-023-01967-0 (PMC9840343; doi:10.1186/s12875-023-01967-0)
Supplement: Supplementary file 1 — Additional file 1: Appendix. Detailed results of qualitative analysis. [file 12875_2023_1967_MOESM1_ESM.docx]

**Appendix: People with early-onset colorectal cancer describe primary care barriers to timely diagnosis: A mixed-methods study of web-based patient reports in the United Kingdom, Australia and New Zealand**

**Appendix: Detailed Results of Qualitative Analysis**

**Domain 1. Clinical assessment**

**Theme 1.1 Provider perception of age as a factor in delay**

A pervasive theme in the discourse on clinical care—and in all three of the patient experience domains—was GPs’ emphasis on the low-risk of CRC for people aged under 50. Symptoms of abdominal distension and pain, bowel changes, anaemia, weight loss, fatigue and anal bleeding and blood in stools that persisted for three months or more were reportedly not investigated for CRC because of age. Patients who presented with classic CRC symptoms and who had a family history of CRC were not screened for cancer because of their age. Colonoscopy referrals were commonly specified as non-urgent because of age. Patients described multiple primary care consultations in which the possibility of cancer was either neglected or explicitly negated as a potential line of clinical inquiry, given their age.

**Theme 1.2 Clinical focus on common conditions**

Patients whose diagnostic journeys were lengthy almost universally described the focus of clinical assessment and management of symptoms as revolving around commonplace and/or chronic conditions. Of these, the most reported diseases were Irritable Bowel Syndrome (IBS) for patients with abdominal pain and bowel changes (all for women), haemorrhoids for patients with bleeding and anaemia or iron deficiency for people with exhaustion, based on blood test results.

A diagnosis, or potential diagnosis, of IBS in our study was nearly always accompanied by investigations for, and diagnoses relating to gynaecological issues such as heavy menstruation and endometriosis as well as pregnancy-related and post-natal conditions. Additionally, only personal accounts written by women reported GP assessments involving potential emotional and psycho-social causes of their physical symptoms. GPs largely focused on stress, particularly for women with children, and on health anxiety related to repeated consultations for symptoms and concern about cancer. Some patients reported being assessed for eating disorders. Again, these patients perceived squandered opportunities for CRC diagnosis.

**Theme 1.3 Adequacy of investigations**

The personal accounts of delay at primary care level heavily featured descriptions of diagnoses of haemorrhoids in the absence of physical examination, multiple investigations for gastrointestinal disorders stopping short of colonoscopy and non-urgent gastroenterologist referrals for significant blood in stools, anal bleeding, bloating, abdominal pain and bowel changes—even when the patient presented with all these symptoms. Patients described repetitive, unproductive investigations that they perceived contributed to delay, including narrow-spectrum blood tests, ultrasounds and dietary allergy testing. Some patients recounted that positive faecal test results and high markers for inflammation highlighted in blood tests were not addressed. These events occurred significantly in relation to primary care but were also noted in emergency care contexts.

**Domain 2. Continuity of care**

**Theme 2.1 Continuity across multiple providers**

Patients reported seeing multiple GPs and having more than one visit to a specialist or emergency care prior to diagnosis. Adequate quality of information in patient records was a key topic; patients reported that their records within GP practices were not always clinically comprehensive or current, and that the records failed to provide an adequate overview of their diagnostic trajectory to date. Patients also described poor information transfer across primary, specialist and emergency levels of care resulting in patients having to redescribe their symptoms and recount treatments and investigations to date with each new provider. Patients expressed concern that information was lost in the retelling and that inadequate records compelled providers to restart lines of investigation, which delayed ultimate CRC diagnosis.

Patients also described contexts in which GPs preferred to repeat investigations undertaken by other GPs. The experience of duplicated investigation was reported across primary providers in different practices but also within the one practice. Patients in the UK in particular reported repetition of tests and treatment as a result of seeing different providers in large, busy primary care practices.

Emergency department visits were conveyed as healthcare events that rarely connected into other parts of the healthcare system. Emergency care was commonly used to manage unbearable abdominal pain, severe diarrhea and vomiting, or extensive bleeding between appointments with general practitioners, or while waiting for specialist appointments. In some seven per cent of all personal accounts, patients described attending emergency care between two and five times before being admitted for further investigations or referred for outpatient colonoscopy. Patients who went to emergency departments described a range of experiences in which they were provided treatment but not offered further investigation.

**Theme 2.2 Referral delays**

Patients perceived that GP referral practices contributed to the circuitous nature of their diagnostic trajectories, especially when GPs continued to make referrals for dietary, gynaecological and mental health investigations as symptoms continued or worsened. Patients also described emergency care physicians and to a lesser extent, gastroenterologists, refusing to refer them for cancer investigations because of their age. The multiple specialist and test appointments and time intervals involved in these referrals were perceived as delays to the eventual CRC diagnosis. Additionally, patients described being given referrals to specialists and colonoscopy screening only when they declared they had private insurance or were willing to pay privately.

**Theme 2.3 Wait times for colonoscopy**

The interval between making the appointment and attending the colonoscopy was of great concern to patients who had been given non-urgent colonoscopy referrals. Some patients identified that they had not been told their referral was non-urgent or that an urgent referral was possible. Patients who had spent long periods of time seeking resolution for their symptoms, and who had sufficient financial capacity, commonly paid privately to be seen sooner for a colonoscopy or reported frustration that they had not been told they could pay privately. Long colonoscopy timeframes were perceived as both a failure of primary care providers to make appropriate referrals, and a breakdown in health system continuity. Patients expressed concern that their cancer may have been found sooner had they received a timely colonoscopy.

**Domain 3. Interpersonal care**

**Theme 3.1 Tension over symptom seriousness**

A common refrain in the discourse on delay was not being listened to by GPs about the intensity and/or persistence of symptoms and pain, and lack of regard for concerns about the possibility of colorectal cancer. Patients reported initially accepting their doctor’s rejection of cancer as a differential diagnosis, but continued emphasis on evidence-based low risk of cancer was perceived as lack of individualised care. In retrospective reflections on their diagnostic experiences, patients expressed resentment and anger about what they perceived as lost opportunities for accurate diagnosis had GPs listened to their concerns. They reported regret that they accepted misdiagnoses and continued with futile treatment plans because of the trust they placed in their doctor’s expertise. Patients *s*poke extensively of a need to change the mindset of healthcare professionals at all levels with regards to collaborative diagnostic care.

**Theme 3.2 Patient self-efficacy**

Patients recounted that the events of lengthy diagnostic journeys had a temporary or ongoing impact on their self-efficacy, including increasing doubt about the validity of their symptoms, escalating anxiety and a diminishing sense of agency in their own care. Patients who were uncomfortable speaking with GPs about their frustrations and concerns reported that tensions over symptom seriousness compromised their confidence to continue to seek help from primary care and, to a lesser extent, from specialist providers. They relied on the anonymity of emergency care services to manage pain and obtain a colonoscopy, which was not often successful. These patients also reported the challenges of conveying their personal circumstances of long, fractured diagnostic trajectories to new GPs and expressed fear that this knowledge was crucial in contextualising providers’ decisions. In their reflections on their overall cancer experiences across all stories of delay, patients reported regret that they forfeited self-efficacy; they used postscripts to their personal accounts to encourage readers to take a proactive stance early in the diagnostic process if they are dissatisfied with the experience of their care.

**Theme 3.3 Reassurance referrals**

As symptoms continued without resolution and initial diagnoses appeared to be invalid, patients became frustrated by GPs’ verbal assurances that their symptoms were not serious. They requested cancer screening, usually with colonoscopy*,* to assure them they did not have CRC or some other form of cancer. The time interval before patients requested cancer screening varied. GPs who responded to referral requests despite their own confidence of the low risk for cancer were explicit about their actions as a function of reassurance. Patients commonly reported that reassurance referrals to gastroenterological care and for colonoscopy screening or other highly predictive form of imaging were turning points in their relationships with GPs and in their diagnostic trajectories, leading directly to the finding of cancer.
